# Two novel missense mutations in the myostatin gene identified in Japanese patients with Duchenne muscular dystrophy

**DOI:** 10.1186/1471-2350-8-19

**Published:** 2007-04-12

**Authors:** Atsushi Nishiyama, Yasuhiro Takeshima, Kayoko Saiki, Akiko Narukage, Yoshinobu Oyazato, Mariko Yagi, Masafumi Matsuo

**Affiliations:** 1 Department of Pediatrics, Kobe University Graduate School of Medicine, Kobe, 6500017, Japan

## Abstract

**Background:**

Myostatin is a negative regulator of skeletal muscle growth. Truncating mutations in the myostatin gene have been reported to result in gross muscle hypertrophy. Duchenne muscular dystrophy (DMD), the most common lethal muscle wasting disease, is a result of an absence of muscle dystrophin. Although this disorder causes a rather uniform pattern of muscle wasting, afflicted patients display phenotypic variability. We hypothesized that genetic variation in myostatin is a modifier of the DMD phenotype.

**Methods:**

We analyzed 102 Japanese DMD patients for mutations in the myostatin gene.

**Results:**

Two polymorphisms that are commonly observed in Western countries, p.55A>T and p.153K>R, were not observed in these Japanese patients. An uncommon polymorphism of p.164E>K was uncovered in four cases; each patient was found to be heterozygous for this polymorphism, which had the highest frequency of the polymorphism observed in the Japanese patients. Remarkably, two patients were found to be heterozygous for one of two novel missense mutations (p.95D>H and p.156L>I). One DMD patient carrying a novel missense mutation of p.95D>H was not phenotypically different from the non-carriers. The other DMD patient was found to carry both a novel mutation (p.156L>I) and a known polymorphism (p.164E>K) in one allele, although his phenotype was not significantly modified. Any nucleotide change creating a target site for micro RNAs was not disclosed in the 3' untranslated region.

**Conclusion:**

Our results indicate that heterozygous missense mutations including two novel mutations did not produce an apparent increase in muscle strength in Japanese DMD cases, even in a patient carrying two missense mutations.

## Background

Duchenne muscular dystrophy (DMD), the most common inherited myopathy affecting approximately one in 3,500 males, is characterized by muscle dystrophin deficiency. Dystrophin deficiency is caused by translational reading frame shifts or nonsense mutations in the dystrophin gene [1]. DMD is a rapidly progressive disease occurring during childhood that causes affected individuals to lose their ability to walk by the age of 12 years old before they succumb during their twenties due to either respiratory or cardiac failure.

DMD is known to progress with a rather uniform pattern of muscle weakness, However, the existence of a modifying gene has been suggested due to the identification of unusually mild DMD phenotypes [2-4]. Some phenotypic variability has been explained by the precise locations of the mutations and their effects on the dystrophin-dystroglycan complex [5,6] or by the identification of aberrant splicing products from the dystrophin gene [7-10]. In some cases, however, the same dystrophin mutation has been reported to result in different phenotypes [11,12].

Although some phenotypic variability may arise due to environmental factors, such as diet or exercise, there are likely to be contributions from genetic components. In fact, differences in genetic backgrounds have been shown to influence the phenotypes of mice with a dystrophin-glycoprotein complex disorder caused by a mutation in the σ-sarcoglycan gene [13]. Therefore, it is highly plausible that unknown genetic factors modify the phenotype of DMD.

Myostatin, also known as growth and differentiation factor 8 (GDF8), is a muscle-specific secreted peptide that functions to limit muscle growth [14]. Several studies analyzing mutations of the myostatin gene have been conducted in the Western worlds [15-17]. To date, six polymorphisms and one intronic mutation have been identified in the myostatin gene. One of the identified polymorphisms (p.153K>R) has been associated with a hypertrophic response in muscles due to strength training [18]. Recently, an infant was identified with the first homozygous disruption of the myostatin gene, which resulted in the child being exceptionally muscular at birth and unusually strong with increased muscle mass at four years of age [19]. Remarkably, a single nucleotide change creating a potential illegitimate micro RNA target site in the 3' untranslated region of the sheep myostatin gene was disclosed to cause translation inhibition leading to the increase of muscularity [20].

Furthermore, disruption of endogenous myostatin by gene or RNA targetings was shown to result in anatomic, biochemical, and physiologic improvements in the dystrophic phenotype of *mdx *mice, a mouse model of DMD with a nonsense mutation in the dystrophin gene [21,22], including particularly prominent enlarged fiber diameters and greatly reduced fatty fibrosis. These results suggest that blocking endogenous myostatin is a potential strategy for treatment of DMD [23].

We hypothesized that genetic variation in the myostatin gene modifies the phenotype of DMD. Therefore, nucleotide changes in the myostatin gene were investigated in Japanese DMD patients, resulting in the identification of novel mutations.

## Methods

### Subjects

One hundred two DMD patients that were followed up at Kobe University Hospital were enrolled into this study. All of the mutations in the dystrophin genes were revealed to introduce premature stop codons in the dystrophin mRNA; 51 cases with mutations that induced a translational reading frame shift due to exon deletion or duplication, 31 cases with nonsense mutations, 12 cases with mutations of one or a few nucleotides deletion or insertion, and 8 cases with intron mutations that induced splicing error (data not shown). The subjects' ages ranged from 1 to 31 years old (average: 10 years old). Regular clinical check-ups, including determination of the serum creatine kinase (CK) levels, were performed at the outpatient clinic. The maximal voluntary isometric torque (MVIT) produced by the elbow flexor muscles and the knee extensor muscles was measured with a manual dynamometer (Microfet2 digital muscle tester, Value Medical Supplies, Hesperia, CA) with a precision of 0.1 Nm. A clear difference in the phenotypes was observed in the ages at which the patients became wheelchair bound, which occurred between the ages of 5 and 11 years old. Some patients, however, were able to walk independently after they were 12 years old even though they carried mutations that caused truncations of the dystrophin protein.

Protocols of this study were approved by the ethics committee of the Kobe University School of Medicine. Blood samples were taken after written informed consent was obtained.

### Sequencing analysis of the myostatin gene

**Figure 1:**
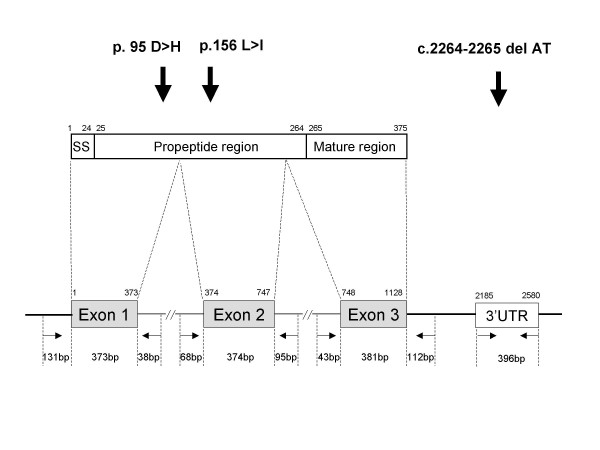
**Myostatin and the human myostatin gene**. The structure of myostatin, including the signal sequence (SS), and the regions of the propeptide and mature protein, is schematically described (Top). The numbers above the boxes indicate the amino-acid residue position. The vertical arrows indicate the locations of the two novel missense mutations and two nucleotides deletion identified in this study. The structure of the myostatin gene and the analyzed regions are schematically described (Bottom). Three coding regions (Exons 1, 2 and 3) and a part of the 3' untranslated region (3'UTR) of the myostatin gene were PCR amplified (boxes). The shaded and open boxes indicate the coding region and the sequenced region in the 3' untranslated region, respectively. Bold and thin horizontal lines indicate exons and introns, respectively. Horizontal arrows indicate the locations and directions of the primers used to amplify the regions. Numbers above the boxes indicate the nucleotide position according to the cDNA reference sequence in GenBank (accession no.: NM_005259), in which the "A" in the start codon is nucleotide #1. Numbers in the bottom indicate the size of each segment.

Genomic DNA samples were prepared from the peripheral blood of the patients via the standard phenol-chloroform extraction method and were used as templates for PCR amplification. All three of the myostatin exons were examined by PCR amplification and direct sequencing (Fig. 1). Exons 1 and 3 of the myostatin gene were PCR amplified as previously described [19]. Exon 1 was amplified as a 542-bp fragment, including 131 and 373 bp of the 5' untranslated and protein coding regions, respectively, as well as 38 bp of intron 1 (Fig. 1). Exon 3 was amplified as a 536-bp fragment including 43 bp of intron 2 and 381 and 112 bp of the protein coding and 3' untranslated regions, respectively (Fig. 1). Additionally a middle part of the 3' untranslated region was amplified as 396-bp fragment using a set of two primers (3UF: 5'-CATGTCATGCATCACAGAAAAGCAACTACT-3' and 3UR: 5'-CAAAATCCCAATTTACAAAACAGAA-3'), since a single nucleotide change in this region of the sheep myostatin gene has been shown to create a microRNA target site, thereby leading translation inhibition [20]. Exon 2 was amplified using two primers (2F: 5'-ATTAATATGGAGGGGTTTTGTTAATGG-3', 2R: 5'-GCTTAGGGAATTTGTAGCTATTTTCCA-3') that resulted in a 537-bp fragment, including the 374 bp of exon 2, and 68 and 95 bp of introns 1 and 2, respectively.

Denaturation of the DNA was performed at 96°C for 5 min, followed by 35 cycles of denaturation at 96°C for 1 min, annealing at 60°C for 1 min, and elongation at 72°C for 1.5 min. The amplified products were analyzed on a 2% agarose gel and visualized by ethidium bromide staining. The PCR-amplified products were directly sequenced using a BigDye Terminator v1.1 Cycle Sequencing kit (Applied Biosystems, Foster City, CA) and an automated DNA sequencer (ABI Prism 310 Genetic Analyzer; Perkin Elmer Applied Biosystems). For subcloning sequencing, the PCR-amplified products were cloned into the pT7 blue T vector (Novagen, Madison, WI) and sequenced. Sequencing results were compared with the wild-type sequence (Genbank: AC073120).

## Results

**Figure 2:**
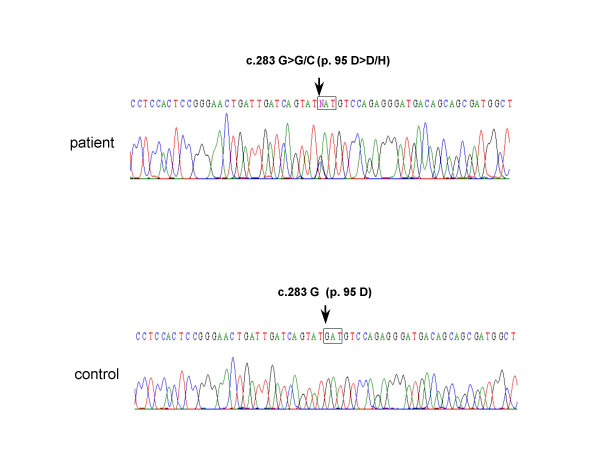
**A novel mutation in exon 1 of the myostatin gene**. A part of the sequencing results for exon 1 of the myostatin gene from a DMD patient (case 712) is shown (patient). Overlapping G and C peaks are present at the 283^rd ^nucleotide of the myostatin cDNA (c.283G>G/C) (Top). The single nucleotide change from G to C at the 283^rd ^nucleotide of the myostatin cDNA (c.283G>C) changed a GAT codon to a CAT codon at the position corresponding to the 95^th ^amino-acid residue of myostatin (p.95D>H)(boxes). The wild type sequence is shown below (control).

All three coding regions and a part of the 3' untranslated region of the myostatin gene were successfully PCR amplified from genomic DNA; 102 DNA samples were subjected to direct sequencing. Sequencing results of the exon 1-encompassing region disclosed completely normal sequences in all of the samples except for one nucleotide change in one sample. In this case (case 712), direct sequencing revealed overlapping G and C peaks at the 283^rd ^position of the myostatin cDNA (c.283G>G/C) (Fig. 2). Because this nucleotide position is a G in the wild-type sequence, the presence of a C at this position was determined to be a mutation (c.283G>C).

c.283G>C changed the codon corresponding to the 95^th ^amino-acid residue of myostatin from GAT to CAT, which substituted an Asp residue to a His residue (p.95D>H). This missense mutation was located at a conserved amino-acid residue in the propeptide region (Fig. 1) [24] and was predicted to affect the function of myostatin. Clinical examination of muscle strength, however, failed to reveal a clear difference between the DMD patient carrying this nucleotide change and the other DMD patients. At 8 years old, the patient could not stand up by himself, but was able to walk independently with a waddling gait.

**Figure 3:**
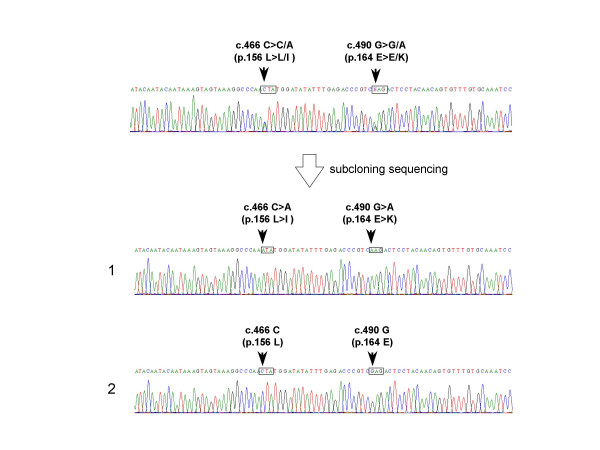
**A novel mutation in exon 2 of the myostatin gene**. A part of the sequencing results for exon 2 of the myostatin gene of one DMD case (case 549) is shown. Overlapping peaks were observed at two locations corresponding to c.466C>C/A and c.490G>G/A in one DNA sample (Top). Subcloning sequencing disclosed two different sequences: one had a completely normal sequence (Bottom 2), whereas the other one had two nucleotide changes (Bottom 1). The G to A change at the 490^th ^nucleotide of the myostatin cDNA (c. 490G>A) matched with the previously described p.164E>K mutation (box). The other nucleotide change from C to A at the 466^th ^nucleotide of the myostatin cDNA (c.466C>A) changes a CTA codon for Lys to a ATA codon for Ile at the position corresponding to the 156^th ^amino-acid residue of myostatin (p.156L>I) (box).

In the exon 2-encompassing region, overlapping G and A peaks at the 490^th ^nucleotide of the myostatin cDNA (c.490G>G/A) were uncovered in four samples. c.490G>A corresponded to a known polymorphism that changes a GAG codon for Glu to a AAG codon for Lys at the position corresponding the 164^th ^amino-acid residue of myostatin (p.164E>K) [15]. The allele frequency of c.490G>A was 2.0% (4 of 204 alleles). Interestingly, in one of the four samples with c.490G>A (case 549), overlapping C and A peaks were also found at the 466^th ^nucleotide of the myostatin cDNA (c.466C>C/A) (Fig. 3). Subsequent subcloning sequencing disclosed the presence of two clones; one was identical to the wild-type sequence (Fig. 3), whereas the second clone carried the two nucleotide changes observed with direct sequencing: c.466C>A and c.490G>A (Fig. 3). c.466C>A, which has not been previously described, changed a CTA codon for Leu to a ATA codon for Ile (p.156L>I). Because the novel missense mutation was located at a conserved residue, this mutation was predicted to disrupt myostatin function (Fig. 1), particularly when it was combined with the second amino acid substitution located only eight amino-acid residues away. Clinical examination of the patient carrying the allele encoding two nucleotide changes did not disclose a clear mild DMD phenotype in the 14 year old. He was wheel-chair bound at 7 years old. The phenotypes of the other three DMD cases carrying c.490G>A in one allele were clinically indistinguishable from the other DMD patients.

None of these Japanese DMD cases carried p.55A>T or p.153K>R, frequently observed polymorphisms in the United States, or the single nucleotide change in intron 2 that has been reported to result in gross muscular hypertrophy (Table 1) [15]. Moreover, examination of the exon 3-encompassing region (Fig. 1) did not disclose any nucleotide changes in the genomic samples. This was compatible with results obtained in previous studies.

**Table 1:** Polymorphisms in the myostatin gene

**mutation**	**Japan**	**USA Caucasian**	**USA African American**	**Italy**	**Belgium**
	**(n = 102)**	(n = 167*) (n = 95**)	(n = 96*) (n = 93**)	(n = 450*) (n = 120**)	**(n = 57)**
p.55A>T	0	12 (het)	19 (het) 2(hom)	2 (het)	0
p.153K>R	0	7 (het)	24 (het) 3 (hom)	6 (het) 1 (hom)	1

*n *number of individuals studied (* p.55A>T, **p.153K>R)

Data are from the USA, Italy, and Belgium [15-17, 27].

**Figure 4:**
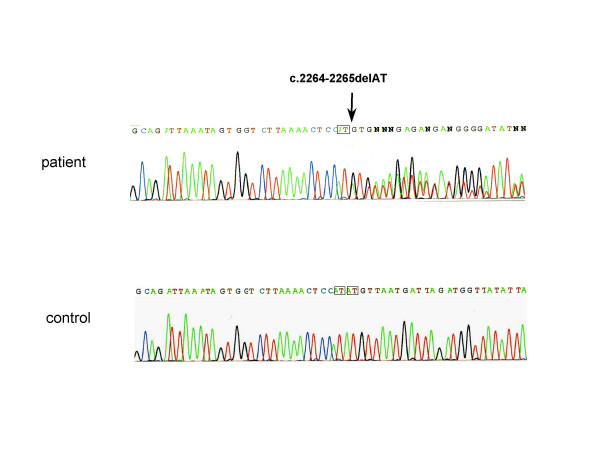
**A novel deletion mutation in the 3' untranslated region of the myostatin gene**. A part of the sequencing result for the 3' untranslated region of the myostatin gene is shown. In the control all of the sequences matched with that of the wild-type sequence (Genbank: AC073120), including the repeat of AT dinucleotides (boxes) (control). In the sequence of one DMD case (case 100) the repeat of AT dinucleotides was not present, removing AT dinucleotides at 2264 and 2265^th ^nucleotide (c.2264-2265delAT) (patient).

Sequencing of the 396-bp fragment of the 3' untranslated region revealed no nucleotide change, especially in the stretch of ACGTTCCA (an underlined G is 2402nd nucleotide where the substitution of G with A has been reported to create the octamer motif for the micro RNA target site in the sheep myostatin gene [20]). Exceptionally, one DMD case (case 100) was found to have a deletion of AT dinucleotides at 2264 and 2265^th ^position (c.2264-2265delAT)(Fig. 4). Though the possibility for the deletion to create the motif for the microRNA target site was searched in this deletion sequence, no candidate motif for the microRNA target site was pointed out [25]. Therefore this deletion seemed a polymorphism in the 3' untranslated region.

## Discussion

Myostatin is a negative regulator of muscle growth that is attracting attention as a novel target for increasing muscle growth in cases of DMD [23]. In this study, we conducted extensive sequence analysis of the myostatin genes in 102 Japanese DMD patients. As a result, two novel missense mutations (p.95D>H and p.156L>I) were identified (Figs. 2 and 3). In addition, a known polymorphism (p.164E>K) was identified in four of the DMD patients. Because one of the DMD patients carried p.156L>I and p.164E>K in the identical allele (Fig. 3), the overall mutant allele frequency was 2.5% (5 of 204 alleles). No truncation mutations in the myostatin gene, however, were identified in our study. In particular, the intron mutation that introduces a premature stop codon in myostatin mRNA resulting in marked muscle hypertrophy [19,26] was not observed. Although a single nucleotide change in the 3' untranslated region of the sheep myostatin gene was shown to lead translational inhibition [20], any nucleotide change creating the octamer motif for the micro RNA target site was not disclosed in DMD patients. Exceptionally, one case was disclosed to harbor two nucleotides deletion in the 3' untranslated region (c.2264-2265delAT) in one myostatin gene (Fig. 4). It needs further study to clarify the meaning of this deletion.

Both of the novel mutations (p.95D>H and p.156L>I) were predicted to be pathogenic because they are located at conserved amino-acid residues at the propeptide region of myostatin [24]. The phenotypes of the DMD cases heterozygous for p.95D>H, p.164E>K, or p156L>1 and p164E>K, however, were not significantly different from the phenotypes of the other DMD cases. It has been reported that a mother carrying a truncation mutation in intron 2 of the myostatin gene appeared muscular, whereas her son, who was homozygous for the same mutation, showed remarkable muscle hypertrophy [19]. Therefore, the muscle volume and strength of individuals that are heterozygous for myostatin mutations may not be markedly affected by these mutations. Considering that women with one missense polymorphism in the propeptide region of myostatin exhibited increase in muscle volume in response to strength training [18], it is supposed that the case with two amino acid substitutions can be phenotypically modified by providing proper muscle rehabilitation. Future studies will address this supposition.

In order to clarify the roles of the two novel mutations, it may be necessary to identify cases in which the mutations are homozygous. In previous studies, homozygous polymorphisms in the myostatin gene have been reported to cause no clear changes in muscle volume or strength [15,16,27]. In this study, some of the DMD patients had mild phenotypes, such as an ability to walk independently past the age 12 years old (data not shown). Although we hypothesized that in these cases the mild phenotypes were a result of a modifier of the DMD phenotype, these patients did not have mutations in their myostatin genes. Particularly we have reported that aberrant splicing products of the dystrophin gene are a modifier of DMD [8,10].

The variability of the human myostatin gene has been studied in Western countries (Table 1) [15-17,27]. To date, six nucleotide changes (two are common and four are uncommon) have been identified in the myostatin gene (Table 1 and 2). Two polymorphisms, a G to A change at codon 55 in exon 1 (p.55A>T) and an A to G substitution in exon 2 (p.153K>R), were represented in 6.6% and 9.8% of the examined alleles in the USA [15]. In an Italian study [16], the p.55A>T and p.153K>R were identified in 0.2% and 3.3% of the examined alleles, respectively. In a study in Belgium, only one individual from 57 males was found to be heterozygous for p.153K>R [17]. None of the Japanese patients in this study, however, carried p.55A>T or p.153K>R (Table 1). These differences in the incidences of the polymorphisms demonstrate that there is a racial difference in the spectra of these polymorphisms.

**Table 2:** Rare mutations in the myostatin gene in the Western world and Japan

**mutation**	**Japan (n = 102)**	**USA (n = 189)**	**Italy (n = 120)**	**Belgium (n = 57)**
p.95D>H	1	ND	ND	ND
p.156L>I	1	ND	ND	ND
p.164E>K	4	2	0	0
p.185R>T	0	ND	1	ND
p.198P>A	0	1	0	0
p.225I>T	0	2	0	ND
c.747+8G>A	0	1	0	ND
c.2264-2265 del AT	1	ND	ND	ND

*ND *not determined, *n *number of individuals studied

All numbers show individuals with mutation in heterozygote

Data are from the USA, Italy, and Belgium [15-17, 27].

It is remarkable that four of the 102 patients were heterozygous for p164E>K, which corresponds an allele frequency of 2.0% in the Japanese patients (Table 1). The p164E>K allele was identified in only 2 out of 189 individuals in the USA, and was not observed in 120 Italians and 57 Belgians [15-17]. In one of the Japanese DMD patients carrying p.164E>K, a second p.156L>I mutation was found in the same allele (Fig. 3). Considering that p.164E>K was identified in both the Americans and Japanese populations, p.164E>K may be an old polymorphism that originated in a common ancestor of the two populations or the polymorphism is located at hot spot for nucleotide changes.

## Conclusion

The present study, although limited to DMD cases, showed the rare occurrence of mutations in the myostatin gene in Japanese subjects. Our results indicate that heterozygous missense polymorphisms including two novel mutations did not produce an apparent increase in muscle volume or strength in Japanese DMD cases, even in a patient carrying two amino acid substitutions.

## List of abbreviations

DMD: Duchenne muscular dystrophy

## Competing interests

The author(s) declare that they have no competing interests.

## Authors' contributions

AN carried out the molecular genetic studies and drafted the manuscript. YT carried out the clinical and molecular genetic studies. KS and AN participated molecular genetic study. YO and MY participated in clinical examinations. MM conceived of the study, and participated in its design and coordination. All authors read and approved the final manuscript.

## Pre-publication history

The pre-publication history for this paper can be accessed here:



## References

[B1] Monaco AP, Bertelson CJ, Liechti-Gallati S, Moser H, Kunkel LM (1988). An explanation for the phenotypic differences between patients bearing partial deletions of the DMD locus.. Genomics.

[B2] Winnard AV, Klein CJ, Coovert DD, Prior T, Papp A, Snyder P, Bulman DE, Ray PN, McAndrew P, King W, Moxley RT, Mendell JR, Burghes AHM (1993). Characterization of translational frame exception patients in Duchenne/Becker muscular dystrophy.. Hum Mol Genet.

[B3] Prior TW, Bartolo C, Papp AC, Snyder PJ, Sedra MS, Burghes AH, Kissel JT, Luquette MH, Tsao CY, Mendell JR (1997). Dystrophin expression in a Duchenne muscular dystrophy patient with a frame shift deletion.. Neurology.

[B4] Hattori N, Kaido M, Nishigaki T, Inui K, Fujimura H, Nishimura T, Naka T, Hazama T (1999). Undetectable dystrophin can still result in a relatively benign phenotype of dystrophinopathy.. Neuromuscul Disord.

[B5] Beggs AH, Hoffman EP, Snyder JR, Arahata K, Specht L, Shapiro F, Angelini C, Sugita H, Kunkel LM (1991). Exploring the molecular basis for variability among patients with Becker muscular dystrophy: dystrophin gene and protein studies. Am J Hum Genet.

[B6] Suminaga R, Takeshima Y, Wada H, Yagi M, Matsuo M (2004). C-terminal truncated dystrophin identified in skeletal muscle of an asymptomatic boy with a novel nonsense mutation of the dystrophin gene. Pediatr Res.

[B7] Chelly J, Gilgenkrantz H, Lambert M, Hamard G, Chafey P, Recan D, Katz P, de la Chapelle A, Koenig M, Ginjaar IB, Fardeau M, Tome F, Kahn A, Kaplan JC (1990). Effect of dystrophin gene deletions on mRNA levels and processing in Duchenne and Becker muscular dystrophies.. Cell.

[B8] Shiga N, Takeshima Y, Sakamoto H, Inoue K, Yokota Y, Yokoyama M, Matsuo M (1997). Disruption of the splicing enhancer sequence within exon 27 of the dystrophin gene by a nonsense mutation induces partial skipping of the exon and is responsible for Becker muscular dystrophy.. J Clin Invest.

[B9] Ginjaar IB, Kneppers AL, v d Meulen JD, Anderson LV, Bremmer-Bout M, van Deutekom JC, Weegenaar J, den Dunnen JT, Bakker E (2000). Dystrophin nonsense mutation induces different levels of exon 29 skipping and leads to variable phenotypes within one BMD family.. Eur J Hum Genet.

[B10] Tran VK, Takeshima Y, Zhang Z, Yagi M, Nishiyama A, Habara Y, Matsuo M (2006). Splicing analysis disclosed a determinant single nucleotide for exon skipping caused by a novel intra-exonic four-nucleotide deletion in the dystrophin gene. J Med Genet.

[B11] Toscano A, Vitiello L, Comi GP, Galvagni F, Miorin M, Prelle A, Fortunato F, Bardoni A, Mora M, Fiumara A, Falsaperla R, Tomelleri G, Tonin P, Danieli GA, Vita G (1995). Duplication of dystrophin gene and dissimilar clinical phenotype in the same family.. Neuromuscul Disord.

[B12] Sifringer M, Uhlenberg B, Lammel S, Hanke R, Neumann B, von Moers A, Koch I, Speer A (2004). Identification of transcripts from a subtraction library which might be responsible for the mild phenotype in an intrafamilially variable course of Duchenne muscular dystrophy. Hum Genet.

[B13] Heydemann A, Huber JM, Demonbreun A, Hadhazy M, McNally EM (2005). Genetic background influences muscular dystrophy. Neuromuscul Disord.

[B14] McPherron AC, Lawler AM, Lee SJ (1997). Regulation of skeletal muscle mass in mice by a new TGF-beta superfamily member. Nature.

[B15] Ferrell RE, Conte V, Lawrence EC, Roth SM, Hagberg JM, Hurley BF (1999). Frequent sequence variation in the human myostatin (GDF8) gene as a marker for analysis of muscle-related phenotypes. Genomics.

[B16] Corsi AM, Ferrucci L, Gozzini A, Tanini A, Brandi ML (2002). Myostatin polymorphisms and age-related sarcopenia in the Italian population. J Am Geriatr Soc.

[B17] Thomis MA, Huygens W, Heuninckx S, Chagnon M, Maes HH, Claessens AL, Vlietinck R, Bouchard C, Beunen GP (2004). Exploration of myostatin polymorphisms and the angiotensin-converting enzyme insertion/deletion genotype in responses of human muscle to strength training. Eur J Appl Physiol.

[B18] Ivey FM, Roth SM, Ferrell RE, Tracy BL, Lemmer JT, Hurlbut DE, Martel GF, Siegel EL, Fozard JL, Jeffrey Metter E, Fleg JL, Hurley BF (2000). Effects of age, gender, and myostatin genotype on the hypertrophic response to heavy resistance strength training. J Gerontol A Biol Sci Med Sci.

[B19] Schuelke M, Wagner KR, Stolz LE, Hubner C, Riebel T, Komen W, Braun T, Tobin JF, Lee SJ (2004). Myostatin mutation associated with gross muscle hypertrophy in a child. N Engl J Med.

[B20] Clop A, Marcq F, Takeda H, Pirottin D, Tordoir X, Bibe B, Bouix J, Caiment F, Elsen JM, Eychenne F, Larzul C, Laville E, Meish F, Milenkovic D, Tobin J, Charlier C, Georges M (2006). A mutation creating a potential illegitimate microRNA target site in the myostatin gene affects muscularity in sheep. Nat Genet.

[B21] Wagner KR, Liu X, Chang X, Allen RE (2005). Muscle regeneration in the prolonged absence of myostatin. Proc Natl Acad Sci U S A.

[B22] Magee TR, Artaza JN, Ferrini MG, Vernet D, Zuniga FI, Cantini L, Reisz-Porszasz S, Rajfer J, Gonzalez-Cadavid NF (2006). Myostatin short interfering hairpin RNA gene transfer increases skeletal muscle mass. J Gene Med.

[B23] Patel K, Amthor H (2005). The function of Myostatin and strategies of Myostatin blockade-new hope for therapies aimed at promoting growth of skeletal muscle. Neuromuscul Disord.

[B24] Tellgren A, Berglund AC, Savolainen P, Janis CM, Liberles DA (2004). Myostatin rapid sequence evolution in ruminants predates domestication. Mol Phylogenet Evol.

[B25] Xie X, Lu J, Kulbokas EJ, Golub TR, Mootha V, Lindblad-Toh K, Lander ES, Kellis M (2005). Systematic discovery of regulatory motifs in human promoters and 3' UTRs by comparison of several mammals. Nature.

[B26] McNally EM (2004). Powerful genes--myostatin regulation of human muscle mass. N Engl J Med.

[B27] Seibert MJ, Xue QL, Fried LP, Walston JD (2001). Polymorphic variation in the human myostatin (GDF-8) gene and association with strength measures in the Women's Health and Aging Study II cohort. J Am Geriatr Soc.

